# Current understanding of the *Streptococcus bovis*/*equinus* complex and its bacteriophages in ruminants: a review

**DOI:** 10.3389/fvets.2025.1466437

**Published:** 2025-05-23

**Authors:** Seon Young Park, Seongwon Seo, Ji Hyung Kim

**Affiliations:** ^1^Laboratory of Aquatic Biomedicine, College of Veterinary Medicine and Research Institute for Veterinary Science, Seoul National University, Seoul, Republic of Korea; ^2^Veterinary Drugs and Biologics Division, Animal and Plant Quarantine Agency, Gimcheon, Republic of Korea; ^3^Division of Animal and Dairy Sciences, College of Agriculture and Life Science, Chungnam National University, Daejeon, Republic of Korea; ^4^Department of Food Science and Biotechnology, College of Bionano Technology, Gachon University, Seongnam, Republic of Korea

**Keywords:** *Streptococcus bovis*/equinus complex (SBSEC), ruminants, metabolic disorders, bacteriophage, biocontrol

## Abstract

*Streptococcus bovis*/*Streptococcus equinus* complex (SBSEC) comprises eight (sub)species, with several opportunistic pathogenic members. These SBSEC species are associated with metabolic disorders in ruminants, resulting in economic losses to the global livestock industry. Moreover, the emergence of antimicrobial resistance (AMR) in SBSEC strains, particularly against commonly used antibiotics, poses serious concerns to the livestock industry. Therefore, alternative approaches to control SBSEC infections have garnered increased attention, and several applications of bacteriophages (phages) have exhibited promising results. Research on SBSEC and their phages has been limited, particularly in livestock production. However, advancements in molecular techniques and a growing interest in alternative strategies to combat AMR have brought SBSEC and their phages into the spotlight. Molecular techniques, such as whole-genome sequencing, have revolutionized the accurate identification and classification of SBSEC, resulting in the elucidation of their ecological and pathogenic roles. SBSEC-infecting phages exhibit remarkable diversity and potential as biocontrol agents, with phage-derived endolysins offering targeted regulation of the SBSEC populations in the rumen. Despite recent achievements, knowledge gaps exist in understanding phage–host interactions and evaluating the efficacy of phage in biologically relevant models, warranting the integration of *ex vivo*, *in vivo*, and *in silico* approaches. Here, we comprehensively review the current knowledge regarding the taxonomy, AMR characteristics, and diversity of SBSEC, and the potential of SBSEC-specific phages, focusing on recent advances in basic research and biotechnological applications in ruminants. Harnessing the potential of SBSEC-specific phages and their derivatives as innovative solutions should help promote overall animal health and the production of antibiotic-free livestock.

## 1 Introduction

*Streptococcus* (*S*.), a gram-positive lactic acid bacterial genus, is a commensal in the gastrointestinal microbiota of humans and animals and comprises many pathogenic species that cause a variety of infections such as bacteremia (1). In the 1930s, based on their cell wall carbohydrate antigens, the Lancefield classification system divided streptococci into several groups, including Group A (with *S. pyogenes*), Group C (with *S. agalactiae*), and Group D (with *S. bovis*) (2, 3). However, streptococci with variable serotypes remains unclassified using this system, exhibiting heterogeneity (e.g., the viridians group). This group was divided into six subgroups: Mitis, Sanguinis, Mutans, Salivarius, Oralis, and Bovis (4). The taxonomy of the *S. bovis*/*equinus* complex (SBSEC), a group of non-beta-hemolytic and non-enterococcal Lancefield group D bacteria, has been revised over the years. Based on the traditional phenotypic and genotypic classification, this complex currently comprises eight species and subspecies: *S. equinus* (previously *S. bovis*), *S. infantarius* subsp. *infantarius*, *S. lutetiensis* (previously *S. infantarius* subsp. *coli*), *S. gallolyticus* subsp. *gallolyticus*, *S. gallolyticus* subsp. *pasteurianus*, *S. gallolyticus* subsp. *macedonicus*, *S. alactolyticus*, and *S*. *ruminicola* (3). Originating from domesticated animals, including horses and cattle, as well as dairy products, some species of this complex, especially *S. equinus*, *S. lutetiensis*, *S. infantarius* subsp. *infanatrius*, and *S*. *ruminicola*, have been associated with metabolic diseases, such as ruminal acidosis, bloat, and mastitis, in ruminants (5–7). *S. gallolyticus* causes severe infections in humans, including infective endocarditis, the prevalence of which is increasing among the elderly (8, 9). The emergence of pathogenicity in SBSEC isolates, as zoonotic pathogens, has been demonstrated by their resistance to a range of antibiotics, such as macrolides and tetracyclines, which are commonly used to treat livestock and veterinary pathogens (10–12). This increasing antibiotic resistance poses significant challenges in the treatment of SBSEC infections, potentially leading to increased public health risks (13), for instance, resistance to erythromycin and tetracyclines has been reported in *S. gallolyticus* isolates derived from endocarditis patients, complicating treatment outcomes (8, 9). Additionally, the virulence of SBSEC is characterized by its ability to invade host tissues, evade immune responses, and produce toxic substances (7, 12, 13). Nevertheless, the mechanisms behind the transition from the commensal to pathogenic form remain unclear for these SBSEC strains. Hence, alternative approaches are required to prevent and control SBSEC.

Highly specific bacteriophages (phages) can effectively target and infect pathogenic bacteria without harming beneficial microbiota in clinical environments and are potential biocontrol agents (14). Their potential has increased interest in exploring the roles and applications of phages in various microbial ecosystems beyond clinical settings, such as the rumen, which offers a complex anaerobic environment in the digestive system of ruminants. Although rumen harbors a diverse microbial community, including numerous phages, studies on the biological characteristics of ruminal phages are scarce due to the difficulties in culture-based isolation, which is complicated by the anaerobic rumen environment (15, 16). Thus, maintaining strict anaerobic conditions throughout isolation procedures is crucial owing to the oxygen sensitivity of diverse ruminal bacteria that serve as hosts for phage propagation, the complex nutritional requirements of ruminal microorganisms, and the difficulty in preserving phage viability during processing of rumen samples (16, 17). Furthermore, the polymicrobial nature of the rumen environment complicates the isolation of phages targeting specific bacterial species, as phage-host interactions can be affected by the presence of other microorganisms and metabolites (18).

Recent advances in sequencing technologies, such as metagenomics, have facilitated the detection of ruminal phages, unveiling previously undetected individual isolates (15, 19). In particular, phages originating from ruminants and dairy products have predominantly been identified within the *Aliceevansviridae*, *Rountreeviridae*, and *Salasmaviridae* families (formerly *Siphoviridae* and *Podoviridae*), according to the International Committee on Taxonomy of Viruses (ICTV), with those targeting SBSEC being among the most thoroughly investigated (20, 21). Moreover, the potential of SBSEC phages and their functional proteins, such as endolysin, has been demonstrated for therapeutic applications and for advancing phage research that provides new perspectives and insights into the ruminal microbiome (22, 23). Therefore, this review aims to provide an overview of recent advances in SBSEC and their phages with a focus on the following points: (i) classification of SBSEC and prevalence of pathogenic SBSEC strains that exhibit antimicrobial resistance (AMR) and virulence, and (ii) investigation of SBSEC-specific phages as alternatives for controlling potential pathogenic strains and as biotechnological tools within the rumen microbiome to improve knowledge and practical applications in livestock production.

## 2 Methodology for literature search

### 2.1 Search strategy

A comprehensive literature search was conducted to review the SBSEC diversity and their phage characterization in ruminants. The following electronic databases were used: PubMed, Scopus, Wiley online library, Clarivate, medRxiv, bioRxiv, Web of Science, and Google Scholar. These databases were selected for their extensive coverage of peer-reviewed scientific literature in the fields of animal science, microbiology, veterinary science, and biotechnology. The search included publications from 1963 to 2024 and only in English were considered. The search terms were refined through trial searches based on key terms identified in the publications. The keywords were used alone or in combination to address diverse aspects of SBSEC research, including SBSEC and related species (“*Streptococcus bovis*/*equinus* complex,” “*S. bovis*,” “*S. equinus*,” “*S*. *ruminicola*,” “*S. lutetiensis*,” “*S. infantarius*,” “*S. lutetiensis*,” “*S. gallolyticus*”), antimicrobial resistance and pathogenicity (“antimicrobial resistance,” “virulence factors,” “zoonotic pathogens”), bacteriophage applications (“bacteriophage,” “phage therapy,” “lytic phage,” “temperate phage,” “prophage,” “phage-derived endolysins”), host environment and microbial communities (“rumen,” “ruminal microbiome,” “gut microbiota,” “subacute ruminal acidosis,”), and bioinformatic approaches (“genome sequencing,” “metagenomics,” “phylogenetics,” “taxonomy”).

### 2.2 Inclusion/exclusion criteria

Studies were selected for inclusion criteria in this literature search based on predefined keywords. However, exclusion criteria were applied to publications focusing on non-SBSEC species or unrelated microbial groups. Studying general microbial communities without specific reference to SBSEC or phages were excluded. In addition, non-peer-reviewed articles, editorials, letters, conference abstracts, and freely available online materials such as PhD dissertations and preprints, were not considered for inclusion. Furthermore, the review process focused on peer-reviewed and published studies. The study selection process was conducted in two stages to identify relevant publications. Initially, titles and abstracts were screened to identify potentially eligible studies based on the inclusion criteria. Subsequently, the publications were assessed to confirm eligibility based on the predetermined inclusion and exclusion criteria.

## 3 Ruminal acidosis

**Figure 1:**
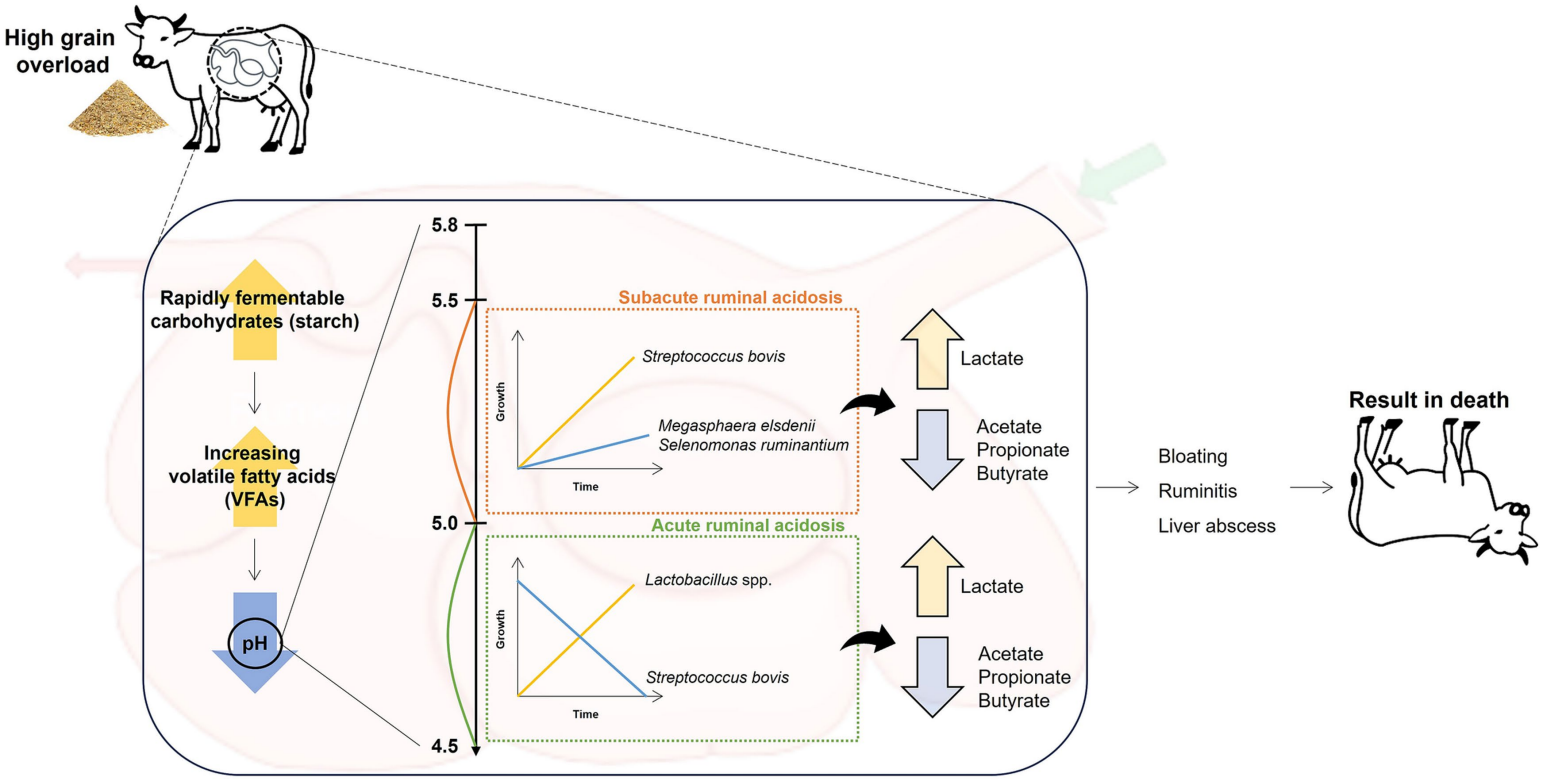
Diagram showing the cascade of ruminal lactic acidosis events.

Ruminal lactic acidosis, a critical metabolic disorder among ruminants reared in intensive farming systems worldwide, can cause serious problems for animal health and productivity, resulting in considerable economic losses (24). In the USA, it has been estimated that the total costs associated with ruminal acidosis in feedlot cattle can range from $10 to $13 per animal, primarily due to reduced feed efficiency and management expenses (24). This disorder occurs when the diet is abruptly changed to include high levels of fermentable carbohydrates and low levels of fibers, causing an influx of non-fibrous carbohydrates in the rumen (Figure 1) (25–27). The dietary alterations lead to the accumulation of volatile fatty acids (VFAs) and lactate, with a reduction in ruminal pH, causing ruminal lactic acidosis (27). This acidification disrupts the delicate microbial balance of the rumen environment in several ways: it inhibits the growth of fiber-degrading bacteria, reduces protozoa populations, impairs rumen motility, and damages the ruminal epithelium (28, 29). Based on the degree of pH reduction, ruminal acidosis is classified as acute and subacute ruminal acidosis (SARA), resulting in a significant shift in the ruminal bacterial composition (26). SARA is characterized by a moderate decrease in ruminal pH to 5.0 and 5.5 that leads to a rapid increase in the population of L-lactic acid-producing gram-positive bacteria, such as SBSEC, which rapidly ferment carbohydrates to lactic acid in the rumen (6, 30). In addition, the shift in several bacterial populations during SARA represents a gradual decline in the abundance of fibrinolytic bacteria, such as *Ruminococcus albus* and *Fibrobacter succinogenes*, which are less tolerant of the lower pH (31). Concurrently, a slow increase in the abundance of lactate-consuming bacteria, including *Megasphaera elsdenii* and *Selenomonas ruminantium*, is observed, which cannot fully compensate for the increased lactate production (32, 33). In contrast, acute ruminal acidosis involves a more drastic pH reduction (<5.0) in the rumen, causing a significant increase in D-lactic acid-producing bacteria, such as *Lactobacillus* spp., and simultaneously, the growth of SBSEC is gradually decreased (34). In ruminal lactic acidosis, the end products fermented by predominant bacteria in the rumen shift significantly because of changes in the microbial population and their metabolic activities, leading to an increase in lactate levels and a potential decrease in the levels of VFAs, especially acetate, propionate, and butyrate (34–36). Thus, the severity and speed of these shifts can immediately and significantly impact the rumen ecosystem and the overall health of the animal, potentially resulting in clinical symptoms, including reduced food intake and fiber digestion, diarrhea, ruminitis, liver abscesses, and even death (24, 37, 38). These symptoms can directly lead to decreased productivity, such as milk yield in dairy cows and lower weight gain in beef cattle, causing significant economic losses in the livestock industry (37, 38). However, the impact of ruminal lactic acidosis differs markedly in the rumen ecosystem, for example, on ruminal pH levels, associated predominant ruminal bacteria, and clinical symptoms. This underscores the importance of considering these differences when (i) managing dietary transitions, (ii) monitoring ruminal health to address shifts in predominant ruminal bacteria, and (iii) preventing the risks associated with each condition of ruminal acidosis. For instance, the use of rumen pH monitoring devices, such as wireless indwelling sensors or rumen boluses, can provide real-time data on rumen pH fluctuations, enabling early detection of acidosis and facilitating timely interventions (39). Additionally, dietary adjustments, such as the inclusion of buffering agents (e.g., sodium bicarbonate) or the use of strategic feeding practices (e.g., total mixed ration), can help stabilize rumen pH and prevent potential acidosis (40, 41).

## 4 *Streptococcus bovis*/*Streptococcus equinus* complex (SBSEC)

### 4.1 Historical and current taxonomy

Historically, the Lancefield serological system categorized streptococci into several groups based on the carbohydrate composition of their cell walls. This system employed antibodies to detect specific antigens and identified the Lancefield group D antigen as a marker for a subset of *Enterococcus* spp. and certain *Streptococcus* spp., including SBSEC (2, 3). Enterococci were later distinguished from streptococci by their ability to grow in the presence of bile and under high salt concentrations (42, 43). In 1919, *S. bovis* (SB), initially identified as a non-enterococcal Lancefield group D antigen, was isolated from the bovine intestine, where it is implicated in ruminal disorders, including SARA (3, 44). *S. equinus* (SE), originating from equine sources and classified within serologic group D, was later identified as a heterotypic synonym of SB. This identification led to a reclassification within the several species (or subspecies) of SBSEC (11, 45).

**Figure 2:**
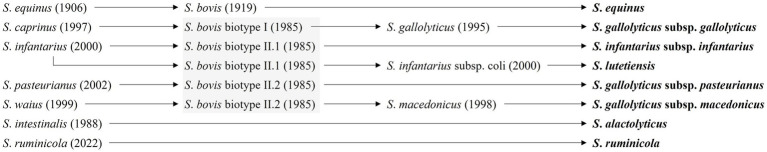
Current and previous nomenclature for SBSEC. The taxonomic overview was adapted from Hinse et al. (60) and the gray boxes depict the classification within the biotypes proposed by Parker and Ball (161).

Although SBSEC species were primarily found in animals and their products, several species originating from human infectious diseases were also discovered with some clinical isolates exhibiting AMR (7, 11). Thus, the necessity for accurate identification at the (sub) species level within SBSEC was emphasized to devise advanced diagnostic approaches that effectively manage infections caused by pathogenic SBSEC isolates (46, 47). The taxonomy of SBSEC has been undergoing revision for the past 20 years, prompting reclassification and identification of new species and subspecies (3). In the 1980s, SBSEC strains were divided into two biotypes based on their ability to ferment mannitol and other carbohydrates, namely biotype I, which could ferment mannitol (*S. gallolyticus* subsp. *gallolyticus*) and biotype II, which could not ferment mannitol; based on the ability to degrade bile-esculin and use it in subsequent biochemical reactions, biotype II was further divided into two subtypes, namely biotype II/1, which could degrade bile-esculin (*S. infantarius* subsp. *infantarius* and *S. lutetiensis*) and biotype II/2, which could not degrade bile-esculin (*S. gallolyticus* subsp. *pasteurianus* and *S. gallolyticus* subsp. *macedonicus*) (Figure 2) (48–50). Despite this phenotypic classification of SBSEC, the complexity of this group necessitates further exploration of phenotypic and genotypic identification methods to accurately distinguish between species and subspecies and to potentially uncover new members within the SBSEC.

### 4.2 Phenotypic and proteomic identification

The main method for identifying SBSEC strains relies on phenotypic characteristics, particularly their hemolytic patterns on blood agar and tolerance to high salt concentrations (51, 52). SBSEC strains typically exhibit the absence of beta-hemolysis (alpha- or no hemolysis) and cannot grow in the presence of 6.5% NaCl, which traditionally differentiates them from closely related genera, such as *Enterococcus* (11). For better discrimination between SBSEC strains, more detailed phenotypic approaches have been developed, incorporating various enzymatic reactions and biochemical tests. These methods include the fermentation of various carbohydrates, such as mannitol, trehalose, inulin, and esculin, which can help differentiate between SBSEC species and subspecies (11, 53). For instance, *S. gallolyticus* subsp. *gallolyticus* (SGSG) is typically mannitol-positive (54), whereas *S. infantarius* (SI) (5) and *S. gallolyticus* subsp. *pasteurianus* (SGSP) (50) are typically mannitol-negative. Furthermore, the activity of specific enzymes, such as beta-glucuronidase, alpha- and beta-galactosidase, and beta-mannosidase, has been utilized to differentiate between SBSEC species (5, 55, 56). SGSP is generally beta-glucuronidase-positive, whereas SGSG is negative. Similarly, SI and SGSP are often positive for alpha- and beta-galactosidase activities, whereas SGSG is negative. The ability to degrade carbohydrates, such as starch and glycogen, has also been used to distinguish among the SBSEC species (5, 50, 51). SI is typically positive for starch degradation, whereas SGSG is negative, and SGSP shows variable results. Glycogen degradation is observed in SGSG and SI, but not in SGSP.

Accurately classifying SBSEC at the species level remains challenging for the traditional identification method. Therefore, commercial identification systems, such as API and matrix-assisted laser desorption/ionization time-of-flight mass spectrometry (MALDI-TOF MS), have been introduced (57, 58). These systems offer improved discrimination and are the most commonly used methodologies for identifying species and subspecies in clinical microbiology laboratories. These methods are essential for the rapid and accurate diagnosis of SBSEC. Representative biochemical testing for SBSEC identification evaluates multiple metabolic characteristics according to each reference database. The API 20 Strep and Rapid ID 32 Strep systems can differentiate between SB biotypes I and II and SE but cannot identify other species and subspecies within the SBSEC (7). The Vitek 2 GPID correctly identified 87% of SBSEC strains at the species level and 67% at the subspecies level (46). However, these biochemical methods may be limited by the variability in biochemical profiles within species and by the potential for atypical reactions.

MALDI-TOF systems provide more rapid and dependable identification of SBSEC species compared to biochemical methods (59). The Bruker Biotyper system correctly identified all SBSEC isolates at the species level but had a lower accuracy rate of 82% at the subspecies level (11). The Vitek MS system could identify SBSEC with 87% accuracy at the species level and 67% accuracy at the subspecies level, except for *S. gallolyticus* subsp. *macedonicus*, which was not included in its database (59).

In summary, at the species level, both biochemical and MALDI-TOF MS methods can reasonably distinguish SBSEC from other streptococci in clinical microbiology laboratories. However, in case of ambiguous results from these two representative methods, molecular methods, such as single gene or genome sequencing, remain the most reliable for definitively identifying SBSEC subspecies, according to the latest SBSEC classification.

### 4.3 Genotypic identification

Single gene-based sequencing is widely used for the accurate identification and differentiation of SBSEC species and subspecies (7, 60). While 16S rRNA gene sequencing exhibits high discriminatory power for most bacterial species, the 16S rRNA gene sequences of closely related SBSEC species, especially within the *S. gallolyticus* (SG) subspecies found in ruminants, show high similarity (>99%), which limits their differentiation at the subspecies level (7, 46). This limitation is particularly relevant when studying originated ruminants-SBSEC strains, where accurate subspecies identification is crucial for understanding their biological roles and pathogenic potential. Alternative housekeeping genes offer better resolution for SBSEC subspecies identification in ruminants compared to the 16S rRNA gene (11). The *sodA* gene, which encodes the manganese-dependent superoxide dismutase, has proven to be a reliable target for identifying SBSEC (60, 61). Comparative studies focusing on SBSEC isolates from ruminants have shown that *sodA* sequencing provides better discriminatory power for SBSEC species and subspecies compared to 16S rRNA gene sequencing. Based on the comparison of identity and coverage values for SBSEC strains available in GenBank, *sodA* exhibits a wide range of identity values (84–99.3%) and high coverage (93.1–100%), indicating its potential for enhanced SBSEC classification (46). Additionally, *groEL*, which encodes a heat-shock protein, has been used for SBSEC identification, providing effective discrimination at the subspecies level (62). The *groEL* gene exhibits a narrower range of identity values (91.9–99.2%) but with consistent 100% coverage, indicating its effectiveness in SBSEC identification (46). Other potential targets, including *gyrB* and *rpoB*, have been proposed for enhanced SBSEC classification (11). Notably, *gyrB*, which encodes the beta subunit of DNA gyrase, shows a wide range of identity values (84–99.3%) and high coverage (93.1–100%), similar to *sodA*, suggesting its potential for differentiating SBSEC species and subspecies (46). The *rpoB* gene, encoding the beta-subunit of RNA polymerase, also exhibits a relatively wide range of identity values (94.5–99.8%) but lower coverage (70.4–98.2%) compared to the other genes (46).

Although single-gene-based methods, particularly *sodA* and *groEL* sequencing, offer improved identification of SBSEC species and subspecies from ruminants compared to phenotypic and proteomic approaches, whole-genome sequencing (WGS) represents the most comprehensive approach for SBSEC classification (11, 46). With increasing technological advances and affordability, WGS is expected to play an increasingly pivotal role in the accurate identification of SBSEC strains in ruminants, enabling the discovery of novel species or subspecies within the SBSEC. The genomes of SBSEC strains available in the GenBank exhibit diverse isolation sources and a wide range of genome sizes. They also show varying features of intrinsic AMR genes and prophages integrated within the bacterial genomes. The genomic features related to these aspects are presented in Supplementary Tables 1–8.

The genome of 435 SBSEC strains currently available in the GenBank comprises 48 SE, 133 SI, 76 *S. lutetiensis* (SL), 62 SG, 46 *S. macedonicus* (SM), 52 *S. pasteurianus* (SP), 14 *S. alactolyticus* (SA), and 4 *S*. *ruminicola* (SR) strains. These strains exhibit genome sizes ranging from 1.0 to 2.7 Mbp. The largest genome size is 2,689,636 bp for SP strain An908 (accession no. JAFBIP01), isolated from a pig, whereas the smallest genome size is 1,033,238 bp in a metagenome-derived SG strain S32M_St_metabat_5 (accession no. JAUNNL01) originating from tapir feces. The SBSEC strains have been isolated from diverse sources, including the gut, feces, and several body sites of humans (51, 63, 64) and a wide range of animals, such as cattle, goats, sheep, deer, horses, dogs, cats, camels, koalas, pigs, chickens, and birds (10, 65–69). These strains have also been isolated from food products, including dairy products, such as milk and cheese, and corn, soil, wastewater, and draining-matting conveyors (64, 70–73). The diversity of isolation sources highlights the adaptability of SBSEC to different niches and its potential for zoonotic transmission. The majority of the SBSEC strains were isolated from the gastrointestinal tract of ruminants, as these bacteria are known to cause SARA in the rumen.

Interestingly, the genomes of some SBSEC strains harbor 12 kinds of plasmids: two in SE (p1_CNU_G6, CP046630.1; plas1, CP075173.1), one in SI (pSICJ18-1, CP003296.1), one in SG (pSGG1, FR824044.1), three in SM (pSMA198, CP119173.1; pSMA198, HE613570.1; p37_1, CP113441.1), one in SP (unnamed, CP136944.1), two in SA (unnamed1, CP114884.1; unnamed2, CP114885.1), and two in SR (p_CNU_G2, CP046920.1; p1_CNU_G3, CP046625.1) (Supplementary Tables 1–8). The presence of these plasmids suggests the potential for horizontal gene transfer (HGT) and acquisition of novel genetic elements, including AMR genes, among SBSEC strains (Supplementary Tables 9, 10) (7, 74).

To investigate the genomic features of SBSEC species, we analyzed the complete genome assemblies of representative strains to identify prophage regions using the PHAge Search Tool Enhanced Release (PHASTER) web server (Supplementary Table 11). Prophages contribute to the acquisition of pathogenic traits in bacteria through mechanisms, such as phage-mediated HGT, AMR genes, and virulence factors (74). The prophage regions varied in length from 11,580 to 67,793 bp and encoded between 11 and 71 proteins. Further investigation of the specific genes and functions associated with these prophage regions could provide valuable insights into the mechanisms underlying the pathogenic potential of SBSEC.

Although single gene-based sequencing, particularly targeting *sodA* and *groEL*, is a robust method for accurate identification and differentiation of SBSEC species and subspecies, WGS has emerged as the most effective approach for SBSEC classification, enabling the discovery of novel taxa within the complex. An extensive collection of SBSEC genomes available in the GenBank has revealed a wide spectrum of isolation sources and genome sizes, along with diverse intrinsic AMR genes and prophage elements integrated within their genomes. Further investigation using advanced sequencing-based technologies, such as metagenomics, particularly gut microbiome analysis, holds immense potential for unraveling the diversity of SBSEC. This approach facilitates the identification of novel species and can lead to the development of effective strategies for their management and control.

## 5 Antimicrobial resistance

### 5.1 Use of antimicrobials and their resistance in livestock: implications for SBSEC

The discovery of antibiotics in the 20th century revolutionized the treatment of bacterial infections (75). However, their extensive use in humans and animals has led to the emergence and rapid spread of antibiotic-resistant bacteria, which now pose a severe threat to global public health (75). This broad pattern of antimicrobial use and resistance is particularly relevant to understanding the emergence of resistant SBSEC strains in ruminants. Within the complex microbial communities of ruminants, SBSEC faces selective pressures from commonly used veterinary antibiotics, leading to the acquisition of resistance. Antibiotics exert a strong selective pressure on bacterial populations, including SBSEC in the rumen, favoring the survival and proliferation of resistant strains. These bacteria can serve as reservoirs of AMR genes, potentially facilitating the acquisition and spread of resistance determinants through HGT or mutations in bacterial genomes that confer reduced susceptibility to antibiotics (76, 77). In the context of SBSEC in the ruminal microbial communities, this provides opportunities for resistance transfer between different *Streptococcus* species and other members of the microbial community.

The use of antibiotics in domestic animals, especially for growth promotion and disease prevention, has come under increasing scrutiny owing to its contribution to the global issue of AMR (78, 79). Considering the growing threat of AMR, several countries have restricted the antibiotics in livestock (80). For instance, the European Union prohibited the use of antibiotics as growth promoters in animal feed in 2006 (81), and South Korea followed suit in 2011 (82). Similarly, in 2017, the United States Food and Drug Administration introduced the Veterinary Feed Directive, which mandates veterinary oversight for the administration of medically important antibiotics in animal feed (83). Despite these efforts, antibiotics, such as tetracyclines, penicillin, macrolides, and cephalosporins, are widely used for disease prevention and treatment in livestock (84, 85). These antibiotics have shown significant levels of resistance in SBSEC isolates (10).

Among these antibiotics, tetracyclines and macrolides exhibit the highest rate of resistance and are commonly used to treat bacterial infections, such as respiratory infections and mastitis in cattle (86), which are often caused by gram-positive bacteria, such as *Streptococcus* (87, 88) and *Enterococcus* species (89, 90). Frequent exposure of ruminal microbial communities to these antibiotics imposes specific selection pressures that contribute to the AMR patterns observed in SBSEC, as detailed in Section 5.2.

The increasing prevalence of AMR is not limited to pathogenic bacterial species; it also encompasses commensal bacteria residing within the gastrointestinal tract of animals (86). These commensal bacteria can act as reservoirs of AMR genes, harboring a diverse array of resistance determinants. Among the most frequently encountered AMR genes in these bacteria, including SBSEC, are those conferring resistance to tetracyclines, such as *tet*(*M*), *tet*(*O*), *tet*(*A*), and *tet*(*B*), which encode either ribosomal protection proteins or efflux pumps (10, 91–93). Additionally, *erm* genes, particularly *erm*(*B*) and *erm*(*C*), mediate resistance to macrolides, lacosamide, and streptogramin B antibiotics through ribosomal methylation (93, 94). These resistance genes are commonly detected in SBSEC isolates from ruminants, as discussed in Section 5.2.

AMR genes can be transferred from commensal to pathogenic bacteria or vice versa through HGT mechanisms, such as conjugation, transformation, and transduction, indicating an exchange of genetic material between these two groups within the gut microbiome (95, 96). This is particularly relevant for SBSEC strains, which can transition from commensal to pathogenic roles under certain conditions, potentially transferring resistance genes between different microbial populations in the rumen. The collection of both known and unknown AMR genes within the gut microbiome, often referred to as the “resistome,” contributes to the emergence of multidrug-resistant pathogens that are increasingly difficult to treat (97).

### 5.2 Antimicrobial resistance in the SBSEC

The emergence of AMR in the SBSEC is a growing concern for both human and animal health, as these bacteria are implicated in various diseases. In humans, SGG is associated with endocarditis and colorectal cancer (98), whereas SGP is linked to biliary tract infections and liver abscesses (99). In ruminants, the overgrowth of SE (or SB) in the rumen can lead to SARA and bloat, causing significant economic losses in the livestock industry (7). In this respect, both commensal and pathogenic SBSEC strains exhibit high rates of resistance to antibiotics commonly used in veterinary medicine, including tetracycline, erythromycin, and clindamycin (85, 86). Moreover, SBSEC isolates are reservoirs of AMR genes and potentially pose the risk of transferring these genes to other bacteria, including pathogenic species, within the gut microbiome (10, 12, 100, 101). To highlight the latest trends and the potential risks associated with AMR in SBSEC, this review provides recent profiles of AMR and the presence of AMR genes in SBSEC isolates, with a particular focus on those originating from ruminants and dairy products (Table 1).

**Table 1:** Current insights into the antibiotic resistance of SBSEC originating from ruminants and its related products.

Antibiotic class	Antibiotics	SBSEC (sub)species	Origin	Country	AMR genes	References
Tetracycline	Tetracycline	*S. equinus*	Mastitis milk	Korea, Taiwan		(104)
			Cattle	Korea	*tet(M)*	(10)
			Cheese	Italy	*tet(S/M)*	(106)
		*S. gallolyticus*	Mastitis milk	Japan	*tet(M), tet(O), tet(L)*	(100)
			Cheese	Turkey		(12)
		*S. lutetiensis*	Mastitis milk	Korea		(103)
			Cheese	Turkey		(12)
MLS_b_	Erythromycin	*S. gallolyticus*	Mastitis milk	Japan	*erm(B)*	(100)
			Cattle	Belgium	*erm(B)*	(101)
			Cheese	Turkey		(12)
		*S. lutetiensis*	Cheese	Turkey		(12)
	Lincomycin	*S. equinus*	Mastitis milk	Korea, Taiwan		(102–104)
			Cattle	Korea	*lnu(C)*	(10)
		*S. gallolyticus*	Mastitis milk	Japan		(100)
	Clindamycin	*S. gallolyticus*	Cattle	Belgium		(101)
Aminoglycoside	Gentamycin	*S. equinus*	Mastitis milk	Korea, Taiwan		(102–104)
	Streptomycin	*S. gallolyticus*	Cattle	Belgium		(101)
	Gentamycin, Streptomycin, Kanamycin	*S. gallolyticus*	Cheese	Turkey		(12)
		*S. lutetiensis*	Cheese	Turkey		(12)
		*S. infantarius*	Cheese	Turkey		(12)
Beta-lactam	Penicillin	*S. equinus*	Mastitis milk	Korea, Taiwan		(102–104)
Cephalosporin	Cefazolin, Cefuroxime, Ceftiofur	*S. equinus*	Mastitis milk	Korea, Taiwan		(102–104)
Glycopeptide	Vancomycin	*S. gallolyticus*	Cattle	Belgium	*vanA*-Tn1546	(101)

#### 5.2.1 Tetracycline resistance

Tetracycline resistance is widely distributed among SBSEC isolated from cattle and dairy products, possibly due to the extensive use of tetracyclines in livestock. The prevalence of tetracycline varies considerably across countries and sample origins. In Korea, studies have reported tetracycline resistance rates of 15.7% (8/51) in SBSEC isolates from domestic ruminants (10), which is relatively lower than isolates of clinical origin. However, a higher resistance rate of 42.9% (3/7) was observed in SL isolated from bovine mastitis milk samples in Korea (102, 103). In Taiwan, tetracycline resistance was dominant among streptococci from mastitis milk, with 86% of isolates showing resistance; specifically, four of SB and on of SE isolates were resistance to tetracycline (104). Similarly, in Japan, tetracycline resistance was prevalent in SG isolates from human patients (15/20), diseased animals (11/15), and healthy broiler chickens (11/16), while significantly lower (1/15) in isolates from healthy mammals (100). Notably, certain SG isolates carried *tet* genes encoding ribosomal protection proteins (*tet*(*M*) and *tet*(*O*)) or efflux pumps (*tet*(*L*)). In Korea, a significant proportion of SE isolates from the rumen fluid of ruminants exhibited tetracycline resistance (15.7%), with 23.5% carrying *tet*(*M*). This gene is often associated with mobile genetic elements, such as Tn916 transposons, which can easily transfer resistance determinants between streptococcal species. Thus, *tet*(*M*) can contribute to spreading resistance within microbial communities, especially those in the gut microbiomes of ruminants. Tetracycline-resistant SBSEC strains have been isolated from cheeses in various countries (12, 105). In Turkey, SL and SG isolates from cheese were found to be tetracycline-resistant, whereas in Italy, SE isolates from cheese harbored a mosaic *tet*(*S/M*) gene (106). The presence of these resistant strains in dairy products raises concerns regarding potential food safety risks and the spread of AMR from animals to humans through the food chain.

#### 5.2.2 Macrolide-incosamide-streptogramin B (MLS_b_) resistance

The MLS_b_ class, which includes macrolides, lincosamides, and streptogramin B, is commonly used in veterinary medicine for the treatment of bacterial infections in ruminants, such as mastitis, calf diarrhea, and respiratory infections (107, 108). Following tetracycline resistance, the macrolide (e.g., erythromycin) and lincosamide (e.g., lincomycin and clindamycin) classes exhibited the highest levels of resistance among SBSEC isolates from ruminants and dairy products (109). Macrolide resistance, particularly erythromycin resistance, has been frequently reported in SBSEC isolates from ruminants and dairy products (10, 102). Erythromycin-resistant SG isolates harboring *erm*(*B*) have been identified in mastitis milk samples from Belgium (101) and Japan (100). The *erm* (*B*) gene, which confers cross-resistance to MLS_b_ antibiotics through ribosomal methylation, leads to the development of multidrug-resistant strains and limits the treatment of infections caused by SBSEC. In Turkey, the presence of erythromycin-resistant SG and SL isolates in cheeses has emphasized the need to control the spread of MLS_b_ resistance in SBSEC from dairy products to safeguard both animal and human health (12). Furthermore, resistance to lincosamide antibiotics, such as lincomycin and clindamycin, has been reported in SE and SG isolates from ruminants in several Asian countries (10, 100, 102, 104). In Korea, *lnu*(C), which encodes lincosamide nucleotidyltransferase, was detected in lincomycin-resistant SE isolated from the rumen fluid (10). The presence of *lnu*(C) in SBSEC isolates from ruminants highlights the potential for the HGT of lincosamide resistance determinants to other bacteria within the gut microbiome.

#### 5.2.3 Resistance to other classes of antimicrobials

Although resistance to tetracycline and MLS_b_ antibiotics is more prevalent among SBSEC isolates from ruminants and dairy products, resistance to other classes of antibiotics, including aminoglycosides, beta-lactams, cephalosporins, and glycopeptides, has also been reported.

Aminoglycoside resistance has been observed in various SBSEC species isolated from ruminants and dairy products. In Korea (102, 103) and Taiwan (104), SE isolates from mastitis milk samples were resistant to gentamicin. SG isolates from cattle in Belgium (101) exhibited resistance to streptomycin. In Turkey (12), SG, SL, and SI isolates from cheese showed resistance to gentamicin, streptomycin, and kanamycin. Interestingly, *Streptococcus* is generally reported to exhibit intrinsic resistance to aminoglycosides, which cannot be transferred horizontally (110). However, further investigation is needed to explore the presence of acquired aminoglycoside resistance determinants in SBSEC isolates from ruminants and dairy products.

Resistance to beta-lactams (penicillin) and cephalosporins (cefazolin, cefuroxime, and ceftiofur) have been reported in SE isolates from mastitis milk samples in Korea (102, 103) and Taiwan (104). These findings highlight the potential for the development and spread of resistance to multiple classes of antibiotics in SBSEC isolates from dairy cattle.

Vancomycin-resistant SG isolates from cattle carried *vanA*-Tn1546, which conferred resistance to this glycopeptide antibiotic (101). The *vanA* gene cluster, typically associated with the Tn1546 transposon, is a major determinant of acquired vancomycin resistance in enterococci. The presence of *vanA*-Tn1546 has not been previously reported in SG strains from cattle, as it suggests the potential for the HGT of vancomycin resistance determinants from enterococci to SBSEC.

The availability of complete genome assemblies for several SBSEC strains has enabled the identification of key AMR genes within the genomes (Supplementary Table 12). The most common AMR genes found across SBSEC strains are that encoding resistance to glycopeptides (*vanY* and *vanT*), tetracyclines (*tet*(*M*)), and MLS_b_ antibiotics (*erm*(*T*)*, lnu*(*B*)*, lnu*(*C*), and *lsa*(*E*)). The intrinsic presence of diverse AMR genes within the genomes of SBSEC species may enable their adaptation and survival in environments with high levels of antibiotic exposure, highlighting the importance of developing effective strategies and alternatives to control this resistance (11).

The increasing prevalence of AMR in SBSEC isolates from ruminants and dairy products, especially against tetracyclines, MLS_b_ antibiotics, and other classes, such as aminoglycosides and glycopeptides, indicates the significance of ensuring antibiotic safety and efficacy in veterinary applications. The presence of acquired resistance genes, such as *tet*(*M*), *erm*(*B*), *lnu*(*C*), and *vanA*-Tn1546, exacerbates the risk for HGT of resistance determinants within the gut microbiome and food chain, emphasizing the need for effective control to mitigate the spread of AMR in SBSEC from ruminants.

## 6 Bacteriophages of SBSEC

### 6.1 General description of phage

Bacteriophages (phages) are viruses that specifically infect and replicate within bacterial cells and are the most abundant biological entities in various environments, with an estimated global population of 10^31^ viral particles (111). They have a relatively simple structure, typically consisting of a protein capsid enclosing a DNA or RNA genome, with many possessing a tail apparatus for host attachment and genome delivery (111). Representative morphologies of phages exhibit that tailed phages in the order Caudovirales are the most prevalent. The classification and naming of the phages are maintained by the Bacterial and Archaeal Subcommittee within the ICTV (112). In the latest ICTV taxonomy release, the phages previously classified under the families *Myoviridae*, *Siphoviridae*, and *Podoviridae* have been reassigned to new families based on various viral properties, such as host bacteria species, virion morphology, life cycle, genome type, and genome similarity (113). The new families include *Herelleviridae*, *Straboviridae*, *Kyanoviridae*, *Peduoviridae*, *Rountreeviridae*, *Salasmaviridae*, *Schitoviridae*, *Chaseviridae*, *Demerecviridae*, *Drexlerviridae*, *Orlajensenviridae*, *Madisaviridae*, *Nobecovirus*, *Winoviridae*, *Atkinsviridae*, *Guelinviridae*, *Duneviridae*, *Pachyviridae*, *Mesyanzhinovviridae*, *Molycolviridae*, *Zierdtviridae*, *Arckerviridae*, *Vertoviridae*, and *Zobellviridae*. However, owing to the complexity of features that contribute to phage taxonomy, their classification is complex and still evolving (114). Recent advances in WGS technologies have revealed the genomic and metagenomic sequences of unknown phages; however, a systematic classification of these phage genomes into the ICTV scheme is not available due to a lack of related biological properties (115).

**Figure 3:**
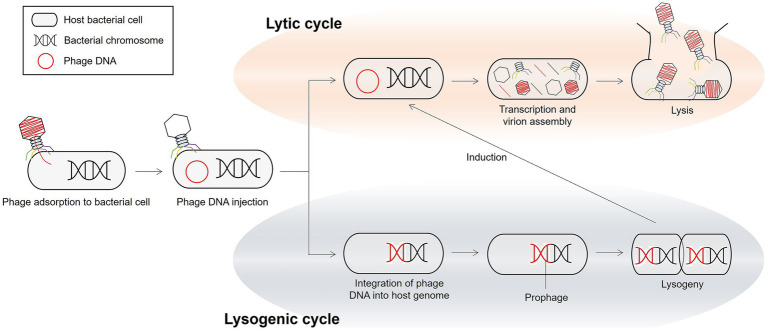
The representative bacteriolytic lifecycles of phages.

Phages have two main life cycles: lytic and lysogenic (Figure 3) (116). In the lytic cycle, virulent phages infect the host cell, take over its metabolic machinery to replicate, and ultimately lyse the cell, releasing progeny virions. Lytic phages are often considered potential biocontrol agents owing to their ability to rapidly multiply and kill bacterial hosts at the end of the replication cycle. In the lysogenic cycle, the genome of a prophage integrates into the chromosome of the bacterial host as a prophage and replicates along with the host until it is induced to enter the lytic cycle. Interestingly, prophages in the lysogenic cycle can horizontally transfer genes, including those for toxins and other virulence determinants, to their bacterial hosts through lysogenic conversion (117). This mechanism significantly impacts bacterial pathogenicity, as many disease-causing toxins, such as those associated with food poisoning, are phage-encoded (118). Moreover, prophages play a role in disseminating AMR genes through phage-mediated transduction and lysogenic conversion (119). Notably, the transfer of resistance genes by phages is often less frequent than through other mechanisms, such as conjugation and transformation (119, 120).

The intricate interactions between phages and their bacterial hosts, as well as the potential applications of phages in various environments, have prompted research into phage populations within specific environments. In particular, ruminants, such as cattle, sheep, and goats, possess a dense microbial community essential for feed digestion in the rumen and for maintaining the overall health and productivity of the animal (121). Investigating the phages associated with the ruminal microbiome can shed light on the dynamics and functionality of the microbial ecosystem within the rumen, offering insights into the potential use of rumen-derived phages and their products for various applications (17).

### 6.2 Ruminal bacteriophages

Bacteriophages are highly abundant in the rumen, with concentrations ranging from 10^7^ to 10^10^ particles per milliliter of rumen fluid (122). Ruminal phages can potentially affect the composition and functionality of the rumen microbiome through phage–host interactions, such as lysis and lysogeny (15, 16). These interactions may lead to enhanced feed digestion efficiency and increased production of metabolites, such as volatile fatty acids, leading to benefit the host animal (115). To date, our understanding of ruminal phages is relatively limited due to the challenges involved in isolating and culturing them. These difficulties arise from several factors, such as the strict anaerobic conditions required to maintain the viability of ruminal phages and their host bacteria, the microbial diversity within the rumen, and the lack of suitable cultivation techniques that can effectively simulate the rumen environment (17). However, recent advancements in sequencing technologies and bioinformatics are expected to contribute to our knowledge of these previously unexplored phages in the rumen (17, 123). Several studies have focused on the isolation and characterization of phages that target specific species of cultured ruminal bacteria (Table 2). These bacteria contribute significantly to diverse aspects of rumen function, such as fiber degradation (*Butyrivibrio fibrisolvens* and *Ruminococcus albus*), lactate utilization (*Selenomonas ruminantium* and *Xylanibacter brevis* (formerly known as *Bacteroides ruminicola ss brevis* and *Prevotella brevis*)), and potential pathogenicity (*Escherichia coli*).

**Table 2:** General features of currently-reported culture-based ruminal phages.

Host bacteria	Phage name	Lifecycle	Isolation source	Morpho-type	Genome		References
					Size (kb)	Accession no.	
*Alkalihalobacillus clausii*	2RFP1E_AA	–	Rumen fluid	–	15	SAMN35002584	(128)
	2RFP1H_AA	Lytic	Rumen fluid	*Myoviridae*	88	SAMN35002585	(128)
*Bacillus safensis*	2RFP5B2_3	–	Rumen fluid	–	41	SAMN35002586	(128)
	3RFP5C_2	Lytic	Rumen fluid	*Siphoviridae*	26	SAMN35002588	(128)
	3RFP5E_6	Lytic	Rumen fluid	*Siphoviridae*	149	SAMN35002589	(128)
	2RFP8A_3	Lytic	Rumen fluid	*Podoviridae*	20	SAMN35002587	(128)
*Butyrivibrio fibrisolvens*	Arawn	Lysogenic	Ruminant^a^	*Siphoviridae*	31	MN882550	(124)
	Arian	Lytic	Ruminant feces	*Siphoviridae*	34	MN882551	(124)
	Bo-Finn	Lytic	Rumen fluid	*Siphoviridae*	33	MN882552	(124)
	Ceridwen	Lytic	Ruminant^a^	*Siphoviridae*	40	MN882553	(124)
	Idris	Lysogenic	Rumen fluid	*Siphoviridae*	31	MN882554	(124)
*Escherichia coli*	P1, P3, P8-P11, P14	Lytic	Ruminant feces	*Myoviridae*	80	–	(129)
	P2	Lytic	Ruminant feces	*Podoviridae*	30	–	(129)
	P6, P7	Lytic	Soil	*Siphoviridae*	35	–	(129)
	1RFP6A	–	Rumen fluid	–	47	SAMN35002581	(128)
	2RFP1A2_1	Lytic	Rumen fluid	*Myoviridae*	88	SAMN35002582	(128)
	2RFP1C2_AA	Lytic	Rumen fluid	*Myoviridae*	135	SAMN35002583	(128)
	TP167CBC_ER F3	Lytic	Rumen fluid	*Myoviridae*	39	SAMN35002591	(128)
*Ruminococcus albus*	Ra01, Ra03	Lytic	Livestock sewage	*Podoviridae*	7, 8	–	(125)
	Ra02, Ra04	Lytic	Livestock sewage	*Inoviridae*	13, 14	JGI1035884, JGI1035887	(20, 125)
*Selenomonas ruminantium*	M-7	Lysogenic	Rumen fluid	*Myoviridae*	30	–	(127)
*Streptococcus bovis*	F4	Lytic	Rumen fluid	*Siphoviridae*	60	–	(136)
*Streptococcus equinus*	Sb01	Lytic	Rumen fluid	*Siphoviridae*	31	JGI1035872	(20)
*Streptococcus ruminicola*	vB_SbRt-pBovineB21	Lytic	Rumen fluid	*Podoviridae*	16	ON759209	(21)
	vB_SbRt-pBovineS21	Lytic	Rumen fluid	*Podoviridae*	17	ON759210	(21)
*Xylanibacter brevis* (formerly *Bacteroides ruminicola ss brevis*)	Brb01, Brb02	Lytic	Sewage	*Siphoviridae*	33, 34	JGI1035879, JGI1035881	(20)
*Xylanibacter brevis* (formerly *Prevotella brevis*)	GA33, B14 phage	Lytic	Rumen fluid	Long-tail	–	–	(126)

a Mixture of rumen fluid and ruminant feces.

Five phages targeting *Butyrivibrio fibrisolvens*, a predominant rumen bacterium involved in fiber degradation and biohydrogenation of unsaturated fatty acids, were isolated from ruminant sources, including feces and rumen fluid (124). These phages are categorized into two distinct life cycles: lytic phages (Arina, MN882551; Bo-Finn, MN882552; Ceridwen, MN882553) and lysogenic phages (Arawn, MN882550; Idris, MN882554). Belonging to the *Siphoviridae* family, these phages have genome sizes ranging from 31 to 40 kb. Also, four lytic phages infecting *Ruminicoccus albus*, which is responsible for the degradation of cellulose in the rumen, were isolated from livestock sewage (20, 125). They were characterized as *Podoviridae* (Ra01 and Ra03) and *Inoviridae* phages (Ra02 and Ra04) with genome sizes ranging from 7 to 14 kb. Previous studies have identified lytic phages (Brb01, Brb02, GA33, and B14 phages) infecting *Xylanibacter brevis* (formerly known as *Bacteroides ruminicola* subsp. *brevis* and *Prevotella brevis*) with a long-tail morphology, indicating their classification within *Siphoviridae* (20, 126). Among these, the genomes of phages Brb01 and Brb02 have been sequenced. These phages infecting bacteria essential for fiber degradation in the rumen could enhance the rate of cellulose and hemicellulose breakdown, thereby affecting the fermentation of forage polysaccharides in the rumen.

A temperate *Myoviridae* phage, M-7, infecting *Selenomonas ruminantium*, was isolated from the rumen fluid of sheep and had a genome size of approximately 30 kb (127). The small size of this phage and its stable lysogenic cycle may serve as the basis of a vector system for the genetic manipulation of *Selenomonas* species, indicating its potential application in the genetic engineering of ruminal bacteria.

Several *E. coli* phages were isolated from ruminant sources, belonging to *Myoviridae* (P1, P3, P8-P11, P14, 2RFP1A2_1, 2RFP1C2_AA, and TP167CBC_ER F3), *Siphoviridae* (P6 and P7), *Podoviridae* (P2), and unclassified families were sequenced (128, 129). Furthermore, the isolation of phages infecting bacterial species that are not typically associated with the rumen microbiome, such as *Alkalihalobacillus clausii* and *Bacillus safensis*, suggests a more diverse phage community than previously appreciated in the rumen (128).

Phages isolated from ruminant sources exhibit considerable diversity based on their bacterial hosts, taxonomy (*Myoviridae*, *Podoviridae*, *Siphoviridae*, and *Inoviridae*), genome size (8–149 kb), and life cycle (lytic and lysogenic). This diversity has significance in rumen environmental function and biotechnological applications. For example, the broad host range exhibited by some *Podoviridae* phages enables them to affect multiple bacterial populations, whereas highly specific *Siphoviridae* phages can target specific strains (20). The diversity in genome size correlates with functional versatility; larger phage genomes often encode auxiliary metabolic genes that can alter host metabolism and biogeochemical cycling within the rumen (130). Additionally, the ratio of lytic to lysogenic phages affects the rate of bacterial turnover and nutrient cycling, with the dominance of lytic phages generally accelerating bacterial lysis and nutrient release (131). Recently, culture-independent isolation and characterization of phages using metagenomic approaches have revealed novel viral sequences in the rumen, many of which cannot be classified into existing taxa. These novel viral sequences include crAssphage-like viruses that serve as modulating populations of Bacteroidetes, which are important for plant polysaccharide degradation in the rumen (132, 133). Additionally, researchers have discovered virus-encoded auxiliary metabolic genes (AMGs) in ruminal viral communities that can potentially modify host metabolism related to sulfur cycling, amino acid synthesis, and carbohydrate utilization, directly causing nutrient fermentation efficiency (134). The vast diversity of uncharacterized ruminal phages potentially leads to the development of phage-based strategies for manipulating the rumen microbiome to improve animal health and productivity, such as targeted biocontrol of methanogenic archaea to reduce enteric methane emissions or enhancement of cellulolytic activity to improve feed conversion efficiency (135).

### 6.3 SBSEC bacteriophages

Bacteriophages targeting the SBSEC have been among the most well-studied ruminant-originated phages due to their potential role in regulating SBSEC populations in the rumen microbiome. In ruminants, SBSEC strains can outgrow other ruminal bacterial flora under optimal growth conditions, producing large amounts of lactate, which can lead to acute ruminal acidosis and bloat (7, 20, 21). This makes SBSEC phages particularly suitable candidates for biocontrol applications in ruminants.

#### 6.3.1 Historical examination of SBSEC phages

The first suggestion of phage therapy for biocontrol in the rumen specifically targeted *S. bovis* (136). Initial studies in the 1970s characterized SBSEC phage isolates from the rumen of cattle and sheep. Iverson and Millis (137) examined the characterization of *S. bovis* phages, establishing foundations about their biological properties. Their research revealed that SBSEC phages exhibited relatively narrow host ranges, primarily infecting specific strains within the SBSEC. Subsequent investigations by Štyriak et al. (138) expanded this study by isolating and characterizing a new ruminal bacteriophage lytic to *S. bovis*. This phage, designated as Sb-1, demonstrated potent lytic activity against multiple *S. bovis* strains, providing early evidence for potential applications in controlling SBSEC populations in the ruminal environment. The representative SB phage F4, belonging to the formerly *Siphoviridae* family, was shown to reduce the adherence of SB strain 47/3 to ruminal epithelial cells, suggesting potential applications for controlling SB colonization in the rumen (136). In parallel, studies on lysogenic SBSEC strains revealed important aspects of phage-host dynamics. Tarakanov (139) investigated the biology of lysogenic strains of *S. bovis* and virulent mutants of their temperate phages. This study demonstrated that lysogeny is common among SBSEC strains in the rumen, with important implications for understanding the ecological relationships between SBSEC and their phages. Further research by Klieve et al. (140) examined the genetic homogeneity and phage susceptibility of ruminal strains of *S. bovis* isolated in Australia, highlighting regional variations in SBSEC phage ecology.

#### 6.3.2 Lytic SBSEC phages: diversity and characterization

Lytic phages that specifically infect SBSEC strains isolated from rumen fluid have been characterized based on their morphological, biological, and molecular properties (Table 3). Gilbert et al. (20) made significant contributions to understanding phage-host interactions in the rumen by sequencing complete genomes of lytic phages infecting rumen bacteria, including SBSEC strains. This study represented a major advancement in the molecular characterization of SBSEC phages and provided insights into their genomic features and evolutionary relationships. Recent advancements have expanded our understanding of SBSEC phages significantly. Park et al. (21) characterized two lytic bacteriophages infecting SBSEC from Korean ruminants, vB_SbRt-pBovineB21 and vB_SbRt-pBovineS21, belonging to the formerly *Podoviridae* family. These phages demonstrated broad-spectrum activity against lactic acid bacteria and exhibited potent antibiofilm properties, suggesting their potential applications beyond the simple lysis of target bacteria. Their work highlighted the diversity of SBSEC phages and their potential utility in controlling SBSEC-associated conditions. Köhne et al. (141) further expanded the known diversity of SBSEC-infecting phages by isolating and characterizing bacteriophages specific to *Streptococcus equi* subspecies *zooepidemicus* and evaluating their efficacy in *ex vivo* models. This study demonstrated the potential for phage therapy applications targeted at specific SBSEC members. Additionally, genomic characterization of SBSEC *Siphoviridae* and *Rountreeviridae* phages isolated from ruminant sources has provided valuable insights into their genome organization and phylogeny (21). SBSEC phages phi-SgaBSJ27_rum and phi-SgaBSJ31_rum, isolated from pigs, revealed remarkably large genome sizes of 110,666 and 106,491 bp, respectively, indicating considerable genetic complexity (142). These findings demonstrate the diversity of SBSEC phage isolation sources and highlight opportunities for discovering novel phages with unique characteristics.

**Table 3:** General features of currently-reported representative lytic SBSEC phages.

Bacterial strain	Phage name	Isolation source	Family	Genome size (bp)	Accession no.	References
*S. bovis*						
47/3	F4	Rumen fluid	*Siphoviridae*	60,380	–	(136)
*S. equinus*						
2B	Sb01	Rumen fluid	*Siphoviridae*	33,595	JGI1035872	(20)
*S*. *ruminicola*						
KCTC 43306	vB_SbRt-pBovineB21	Rumen fluid	*Rountreeviridae*	16,260	MK448367	(21)
	vB_SbRt-pBovineS21	Sewage	*Rountreeviridae*	17,280	ON759210	(21)
*S. gallolyticus*						
BSJ27	phi-SgaBSJ27_rum	Pig	Unclassified	110,666	MN270258	–
BSJ31	phi-SgaBSJ31_rum	Pig	Unclassified	106,491	MN270259	–

The first reported endolysin derived from SBSEC phage, LyJH307, has been characterized biologically and shown to influence the rumen microbiome (22, 23). Endolysins represent an alternative approach to whole-phage applications, potentially offering more precise control over target bacteria. The development of phage-derived enzymes provides additional tools for regulating SBSEC populations in the rumen environment.

#### 6.3.3 Lysogenic and temperate SBSEC phages

In the 1970s and continuing through subsequent decades, researchers have investigated the biological properties of prophages in SBSEC lysogenic strains isolated from the rumen of cattle and sheep. Studies comparing culture conditions between lysogens and virulent mutants of prophages revealed no variance in phage concentration under different conditions (136–138). Even when factors such as glucose, maltose, peptone, or casein hydrolysate were present in the culture medium, these nutrients did not significantly affect phage-host dynamics in SBSEC (139). These findings indicated that SBSEC prophages maintain relatively stable relationships with their bacterial hosts under various environmental conditions. Recent advancements in bioinformatics and genomic analysis have been employed in silico tools to detect prophages and satellite prophages (also known as “helper phages”) in SBSEC genomes (Table 4) (143). A comprehensive analysis of SBSEC genomes revealed 46 phages, including 10 prophages and 13 satellite phages in SE, eight prophages and nine satellite phages in SG, two prophages and one satellite phage in SI, and one prophage and three satellite phages in SL. The genome sizes of these prophages ranged from 27,408 to 45,067 bp, whereas satellite phages exhibited smaller genomes (8,609–13,181 bp). Gilbert and Klieve (16) provided a comprehensive overview of ruminal viruses, including bacteriophages and archaeaphages, emphasizing the ecological significance of prophages in SBSEC populations and their potential contributions to bacterial fitness and adaptation in the rumen environment.

**Table 4:** General features of currently-reported representative putative prophages of SBSEC available in the GenBank database.

Bacterial strain	Phage name	Phage lifecycle	Genome size (bp)	Accession no.
*S. equinus*				
2B	Javan199	Prophage	41,831	MK448702
AG46	Javan200	Satellite	9,586	MK448356
C277	Javan201	Satellite	10,114	MK448357
ES1	Javan202	Prophage	40,975	MK448873
ES1	Javan203	Satellite	10,114	MK448358
GA-1	Javan204	Satellite	9,550	MK448359
H24	Javan205	Satellite	9,984	MK448360
JB1	Javan206	Prophage	37,284	MK448874
JB1	Javan207	Prophage	45,067	MK448703
MPR1	Javan208	Satellite	8,726	MK448361
MPR2	Javan209	Satellite	8,726	MK448362
MPR4	Javan210	Prophage	38,639	MK448875
MPR4	Javan211	Satellite	8,609	MK448364
pR-5	Javan212	Satellite	13,181	MK448365
Sb05	Javan213	Prophage	40,311	MK448704
Sb05	Javan214	Prophage	44,595	MK448876
Sb09	Javan215	Prophage	40,164	MK448705
Sb09	Javan216	Satellite	9,753	MK448366
Sb10	Javan217	Satellite	9,420	MK448367
Sb17	Javan218	Satellite	12,126	MK448368
Sb18	Javan219	Satellite	11,081	MK448369
Sb20	Javan220	Prophage	41,432	MK448877
Ye01	Javan221	Prophage	35,845	MK448706
*S. gallolyticus*				
ATCC_43143	Javan222	Satellite	9,600	MK448371
ATCC BAA-2069	Javan223	Satellite	9,602	MK448372
DD02	Javan224	Prophage	38,781	MK448878
DD02	Javan225	Satellite	9,656	MK448373
DD03	Javan226	Prophage	37,369	MK448879
TX20005	Javan227	Prophage	42,048	MK448707
TX20005	Javan228	Satellite	10,327	MK448374
NTS_31106099	Javan229	Satellite	9,600	MK448375
NTS_31106099	Javan230	Satellite	10,273	MK448376
NTS_31307655	Javan231	Prophage	39,808	MK448709
NTS_31307655	Javan232	Satellite	9,588	MK448377
NTS31301958	Javan233	Prophage	39,808	MK448710
NTS31301958	Javan234	Satellite	9,588	MK448378
UCN34	Javan235	Prophage	45,029	MK448711
UCN34	Javan236	Satellite	9,600	MK448379
VTM3R24	Javan237	Prophage	27,408	MK448712
VTM3R42	Javan238	Prophage	27,408	MK448880
*S. infantarius*				
ATCC BAA-102	Javan263	Prophage	34,637	MK448721
CJ18	Javan264	Prophage	31,478	MK448890
CJ18	Javan265	Satellite	9,627	MK448390
*S. lutetiensis*				
33	Javan284	Prophage	37,997	MK448898
33	Javan285	Satellite	10,789	MK448400
33	Javan286	Satellite	12,433	MK448401
DD06	Javan287	Satellite	9,421	MK448402

#### 6.3.4 Prophages and pathogenicity

To further investigate the potential contribution of prophages to the pathogenicity of SBSEC strains, we conducted an *in silico* analysis of prophage sequences identified in representative SBSEC genomes, as described in Supplementary Table 13. The prophage regions were screened for the presence of pathogenicity-related genes, including AMR genes, virulence factors, and mobile genetic elements (MGEs), using the Comprehensive Antibiotic Resistance Database (CARD^1^), Virulence Factor Database (VFDB^2^), and Mobile Element Finder,^3^ respectively (Supplementary Table 12).

The prophage region of *S. lutetiensis* NCTC 13774 harbored the *vanT* gene, which is part of the *vanG* glycopeptide resistance gene cluster, as well as several virulence factors, such as cytolysin (*cylR2*), serine protease (*htrA*/*degP*), pneumococcal surface antigen A (*psaA*), and streptococcal plasmin receptor/GAPDH (*plr*/*gapA*). These findings suggest that prophages may contribute to the dissemination of AMR genes among SBSEC strains and enhance their pathogenic potential. Moreover, the identification of several insertion sequences (IS) and composite transposons within the prophage regions of SBSEC genomes indicates that prophages can mediate horizontal transfer of MGEs, thereby facilitating the acquisition and dissemination of virulence genes and AMR determinants among SBSEC strains. Transcriptomic analysis of SBSEC strains harboring prophages has shown that these genetic elements may carry genes related to pneumococcal pathogenicity factors, such as *vapE*. The presence of virulence-associated genes in prophages suggests their potential role in the pathogenicity of SBSEC. The acquisition of these genes through prophage integration may contribute to the adaptation and evolution of SBSEC strains in the ruminal environment, potentially enhancing their ability to cause infections in ruminants.

#### 6.3.5 Ecological and evolutionary significance

Phage predation plays a crucial role in regulating dominant SBSEC strains (144), preventing their overgrowth in metabolic disorders such as rumen acidosis. This selective pressure is essential for maintaining microbial diversity, which can be compromised by excessive proliferation of SBSEC. As noted by Laverde Gomez et al. (145), in ruminants, SBSEC strains can outgrow other ruminal bacterial flora under optimal conditions, producing large amounts of lactate and capsular polysaccharide, which leads to acute ruminal acidosis and bloat. Feedlot cattle are commonly fed ionophores (such as monensin and other antibiotics) to increase feed efficiency, limit SBSEC overgrowth, and prevent the subsequent drop in ruminal pH. However, this practice raises serious industrial and public health concerns, highlighting the need for alternative methods of microbial control and ruminal community modulation. Phage predation may lead to negative frequency-dependent selection, whereby phages target the most abundant SBSEC strains, thus preventing them from dominating the population and allowing rarer strains to persist (146). This dynamic contributes to the maintenance of bacterial diversity within the rumen microbiome.

Beyond their lytic activities, phages can also facilitate HGT among SBSEC populations through transduction. Phage-mediated HGT may transfer virulence factors or AMR genes that affect bacterial fitness upon infection of a new host (147, 148). This process can rapidly introduce new genetic material into SBSEC populations, thereby enhancing their genetic plasticity and functional diversity.

Gilbert et al. (17) studied technological advances enhancing current understanding of ruminal phage populations, highlighting that further experimental *in vitro* and *in vivo* studies with viral isolates remain crucial for elucidating the biological properties required for developing effective phage therapies and answering outstanding questions in ruminal phage research.

### 6.4 Application and future prospects of SBSEC bacteriophages

Since the discovery that phages can infect and lyse host bacterial cells, the potential of phage biocontrol as an effective treatment for bacterial infections has been extensively investigated (149). The ubiquitous nature, high specificity, and low inherent toxicity of phages make them a promising alternative to antibiotics, offering a safe and sustainable approach to biological control of human and animal diseases (150). With the increasing prevalence of AMR and global spread of multidrug-resistant bacteria, several studies have focused on the application of phage biocontrol in veterinary medicine, especially for livestock, particularly cattle (149, 150).

One of the earliest clinical trials attempting the treatment of bovine mastitis caused by *Staphylococcus aureus* was conducted in 2006 using the lytic phage K (151). However, the experimental application was not effective in reducing *Staphylococcus aureus*, possibly due to the inactivation or degradation of the phage by the immune response of the mammary gland. In addition to whole phages, phage-derived endolysins have been investigated for their potential in treating bovine mastitis caused by streptococci (152, 153). Endolysins, encoded by phages λSA2 and B30, were characterized and found to exhibit good lytic activity against *S. uberis*, *S. dysgalactiae*, and *S. agalactiae* in milk and a mouse mastitis model.

In 1983, the effectiveness of phage biocontrol in treating pathogenic *E. coli* in calves and lambs with experimental diarrhea, without adverse effects on animals, was investigated (154). The administration of a phage mixture consisting of two phages, P433/1 and P433/2, led to the disappearance of diarrhea within 18–22 h post-treatment. In 1998, a study demonstrated the efficacy of intramuscular injection of phage R in preventing and treating septicemia and meningitis caused by *E. coli* strain K1^+^ in calves, with 100% survival in phage-treated animals (155). In 2006, a bacteriophage preparation called Finalyse targeting *E. coli* O157:H7 received USDA approval for application as a spray or wash on live cattle prior to slaughter to reduce pathogen transfer to meat (156, 157).

*In vitro* biological and genomic characterization of lytic phages (F4, Sb01, vB_SbRt-pBovineB21, and vB_SbRt-pBovineS21) that infect SBSEC has been performed aimed at controlling SARA (20, 21, 136). These phages have shown promising results in targeting and lysing ruminal-originated SBSEC strains, which are major contributors to the onset of SARA. Furthermore, *in vitro* biological characterization and *in silico* studies of the SBSEC phage-derived endolysin LyJH307 validated its ability to control the SBSEC population while minimally affecting the ruminal microbiota (22, 23). The specificity and efficacy of LyJH307 in reducing SBSEC abundance, without significantly altering the overall composition of the rumen microbiome, highlights its potential as a targeted antimicrobial agent.

Thus far, studies on ruminal SBSEC phages have primarily been conducted as *in vitro* experiments based on standard laboratory conditions. To advance this field, it is crucial to consider the following points:

- Verify the applicability and efficacy of phage targeting SBSEC using *ex vivo* and *in vivo* models, particularly in controlling SARA. *Ex vivo* models, such as the rumen simulation technique or a rumen-like bioreactor system, can provide a more realistic representation of the rumen environment while allowing for greater manipulation of experimental conditions (158–160). These models can help bridge the gap between *in vitro* and *in vivo* studies, making them physiologically more relevant. *In vivo* studies will provide valuable insights into the practical application of phage in real-world settings, considering factors, such as phage stability, host specificity, and the dynamic nature of the rumen environment.
- Enhance our understanding of phage functionality and phage–host interactions within the complex rumen microbiome using *in silico* computer modeling (23). *In silico* modeling approaches, such as genome-based metabolic models, can help elucidate the intricate relationships between phages, their bacterial hosts, and the rumen microbiome, enabling the development of more effective and targeted phage-based interventions.

By addressing these gaps, insights obtained from *ex vivo*, *in vivo*, and *in silico* studies can pave the way for the development of phage-based strategies to control SARA and other livestock diseases. With growing interest in sustainable and antibiotic-free animal production, advancements in phage biocontrol studies hold great potential for revolutionizing the management of bacterial infections in cattle and other livestock species, and should ultimately promote animal health and production.

Despite the potential of phage therapy as an alternative to antibiotics in controlling SBSEC, several challenges and limitations need to be addressed for its successful application in ruminants. First, the narrow host range of phages due to their high specificity may necessitate the use of phage cocktails for treating SBSEC infections. Second, lysogeny is another concern, as temperate phages can transmit virulence factors or AMR genes (74), potentially enhancing the pathogenicity of SBSEC. Third, the development of phage resistance in bacteria through various mechanisms, such as inhibition of phage adsorption, restriction-modification system, and CRISPR-Cas system, can limit the efficacy of phage therapy. Studies have shown that repeated exposure of SBSEC strains to phages lead to rapid and spontaneous phage resistance (137, 140). Furthermore, *in vivo* studies have demonstrated the emergence of SBSEC strains with differing sensitivities to phages within the rumen despite the initial absence of phage-resistant bacteria (140). These findings indicate that the complex rumen environment facilitates the frequent occurrence of mutations conferring phage resistance. Addressing these limitations related to phage–host interaction is crucial to the successful implementation of phage therapy for SBSEC control in ruminants.

## 7 Conclusion

SBSEC is a diverse group of commensal bacteria in the gastrointestinal tract of humans and animals. However, certain SBSEC species have emerged as pathogens in ruminants, causing metabolic disorders, such as mastitis, bloat, and SARA. The SBSEC taxonomy has undergone revisions, with the current classification consisting of eight species and subspecies. The accurate identification of SBSEC isolates at the subspecies level is imperative for elucidating the specific roles of different species in disease manifestations, as well as for enabling effective diagnosis and management of various diseases caused by SBSEC. While phenotypic identification of SBSEC strains provides reasonable accuracy at the species level, molecular techniques such as sequencing of genes, such as *sodA* and *groEL*, and WGS offer the most reliable approach for subspecies identification and novel taxa discovery. The emergence of AMR in SBSEC isolates from ruminants and dairy products, especially against commonly used antibiotics, such as tetracycline and MLS_b_, is a growing threat to livestock productivity. Furthermore, the presence of acquired resistance genes, such as *tet*(*M*), *erm*(*B*), and *lnu*(*C*), highlights the risk of horizontal transfer of resistance determinants within the gut microbiome and food chain, emphasizing the need for effective control measures to mitigate the spread of AMR in SBSEC from ruminant sources.

Phages have emerged as promising biocontrol agents against pathogenic and antibiotic-resistant bacteria, including SBSEC strains. The high specificity, low toxicity, and potential of phages as alternatives to conventional antibiotics make them attractive candidates. SBSEC phages isolated from ruminant sources, predominantly belonging to *Siphoviridae* and *Myoviridae*, exhibit diverse morphological, biological, and genomic characteristics, with genome sizes ranging from 31 to 110 kb. Notably, certain lytic phages, such as vB_SbRt-pBovineB21 and vB_SbRt-pBovineS21, that infect ruminant-originated SBSEC strains have demonstrated broad-spectrum lytic activity against lactic acid bacteria and possess anti-biofilm properties. These characteristics suggest their potential as biocontrol agents for controlling SBSEC-related diseases, including economically significant SARA in ruminants. Additionally, the discovery of phage-derived endolysin such as LyJH307 offers novel approaches for specifically regulating SBSEC populations within the complex rumen microbiome, further highlighting the versatility of phage-based applications in livestock production.

Although phage therapy could be a promising alternative to antibiotics for controlling AMR-pathogen infections, such as SBSEC-related disorders in ruminants, it is essential to address its current limitations. The narrow host range, potential for lysogeny, and the emergence of phage-resistant strains are significant challenges that require further investigation. Developing phage cocktails, screening for strictly lytic phages, and exploring strategies to minimize resistance development, such as combination therapies with antibiotics or phage-derived enzymes, could enhance the efficacy of phage therapy.

The identification of prophages integrated within the SBSEC genomes suggests their potential role in the adaptation and evolution of these bacteria in the ruminal environment. Notably, some prophages carry genes associated with virulence factors such as *vapE*, which may enhance the pathogenicity of SBSEC strains upon acquisition. Comprehensive characterization of lytic phages and prophages in SBSEC has significantly broadened our understanding of their genetic diversity and provides novel insights that can aid the development of effective phage-based strategies for controlling SBSEC-related diseases in ruminants.

To further advance the phage therapy in controlling SBSEC, it is crucial to verify the efficacy and applicability of SBSEC phage therapy approaches using *ex vivo* and *in vivo* models, particularly in controlling ruminal acidosis. *In silico* computer modeling techniques can provide insights into phage functionality and phage–host interactions within the intricate rumen microbiome, thereby, facilitating the development of highly optimized phage-based interventions.

This comprehensive review elucidates the current understanding of SBSEC and its bacteriophages in ruminants, highlighting the importance of these microorganisms in animal health, and the potential of phage-based approaches as alternatives to antibiotics. Further studies are needed to elucidate the complex interactions among SBSEC, their phages, and the rumen microbiome and to develop and optimize phage-based strategies for the prevention and treatment of SBSEC-related disorders in livestock. Advancements in genomic and metagenomic technologies will continue to unravel the diversity and roles of SBSEC and their phages in the rumen ecosystem. This enhanced insight will enable the promotion of animal health and productivity while encouraging antibiotic-free and environmentally sustainable livestock production practices.

## Footnotes

^1^ https://card.mcmaster.ca/

^2^ https://www.mgc.ac.cn/cgi-bin/VFs/v5/main.cgi

^3^ https://cge.food.dtu.dk/services/MobileElementFinder/
