# Supplementary material for: Current understanding of the Streptococcus bovis/equinus complex and its bacteriophages in ruminants: a review
Source: Front Vet Sci. 2025 May 23;12:1466437. doi: 10.3389/fvets.2025.1466437 (PMC12141233; doi:10.3389/fvets.2025.1466437)
Supplement: Supplementary file 1 [file Data_Sheet_1.zip › Data Sheet 1/Supplementary Table 11.DOCX]

Supplementary Table 11. Features of the putative prophage region within the genomes of SBSEC strains available in the GenBank database.

| Bacterial strain | Length (bp)^*^ | Position | | Total protein (n) |
| --- | --- | --- | --- | --- |
|  |  | Start | End |  |
| *S*. *equinus* |  |  |  |  |
| MDC1 | 37,377 | 596,137 | 635,514 | 52 |
| *S*. *infantarius* |  |  |  |  |
| FDAARGOS_1019 | 48,728 | 399,246 | 447,974 | 54 |
| *S*. *infantarius* subsp. *infantarius* | |  |  |  |
| CJ18 | 60,039 | 782,947 | 842,986 | 55 |
| *S*. *lutetiensis* |  |  |  |  |
| FDAARGOS_1018 | 44,753 | 1 | 44,754 | 69 |
| NCTC13774 | 42,950 | 111,691 | 154,641 | 64 |
|  | 46,182 | 1,421,660 | 1,467,842 | 71 |
| 033 | 58,424 | 1,333,327 | 1,391,751 | 54 |
|  | 13,265 | 1,937,840 | 1,951,105 | 14 |
| NCTC11436 | 54,778 | 995,631 | 1,050,409 | 66 |
| NCTC8738 | 43,648 | 682,559 | 726,207 | 67 |
| *S*. *gallolyticus* |  |  |  |  |
| NCTC13773 | 49,874 | 485,457 | 535,331 | 67 |
| FDAARGOS_755 | 47,701 | 1,206,169 | 1,253,870 | 62 |
| ICDDRB-NRC-S1 | 37,332 | 1,302,729 | 1,340,061 | 37 |
| UCN34 | 49,054 | 447,428 | 496,482 | 58 |
| *S*. *gallolyticus* subsp. *gallolyticus* | |  |  |  |
| DSM16831 | 49,874 | 485,367 | 535,241 | 62 |
| TX20005 | 47,701 | 404,130 | 451,831 | 62 |
| *S*. *macedonicus* |  |  |  |  |
| CIP105683 | 49,527 | 653,005 | 702,532 | 68 |
| ACA-DC198 | 35,517 | 302,491 | 338,008 | 21 |
|  | 11,580 | 1,329,099 | 1,340,679 | 11 |
| E37 | 21,560 | 1,485,895 | 1,507,455 | 14 |
| *S*. *pasteurianus* |  |  |  |  |
| NCTC13784 | 42,950 | 111,691 | 154,641 | 64 |
|  | 46,182 | 1,421,660 | 1,467,842 | 71 |
| *S*. *alactolyticus* |  |  |  |  |
| LGM | 24,161 | 971,752 | 995,913 | 16 |
| *S*. *ruminicola* |  |  |  |  |
| CNU_G2 | 67,793 | 188,158 | 255,951 | 64 |

^*^PHAge Search Tool Enhanced Release (PHASTER, https://phaster.ca/) web server was used to detect prophages in the complete genome sequence of representative SBSEC strains available in GenBank.
