# Supplementary material for: Current understanding of the Streptococcus bovis/equinus complex and its bacteriophages in ruminants: a review
Source: Front Vet Sci. 2025 May 23;12:1466437. doi: 10.3389/fvets.2025.1466437 (PMC12141233; doi:10.3389/fvets.2025.1466437)
Supplement: Supplementary file 1 [file Data_Sheet_1.zip › Data Sheet 1/Supplementary Table 13.DOCX]

Supplementary Table 13. Features of antibiotic resistance genes present within the genomes of SBSEC strains available in the GenBank.

| Bacterial strain | AMR family^*^ | Drug class | Resistance mechanism |
| --- | --- | --- | --- |
| *S*. *equinus* |  |  |  |
| colony399 | vanY, vanT | Glycopeptide | Antibiotic target alteration |
| CNU_77-23 | vanY | Glycopeptide | Antibiotic target alteration |
|  | qacJ | Disinfecting agents and antiseptics | Antibiotic efflux |
|  | tet(M) | Tetracycline | Antibiotic target protection |
|  | lnu(C) | Lincosamide | Antibiotic inactivation |
| CNU_G6 | tet(M) | Tetracycline | Antibiotic target protection |
|  | qacJ | Disinfecting agents and antiseptics | Antibiotic efflux |
|  | vanY | Glycopeptide | Antibiotic target alteration |
| FDAARGOS_251 | tet(M) | Tetracycline | Antibiotic target protection |
|  | ermB | Macrolide, lincosamide, streptogramin A, B | Antibiotic target alteration |
|  | vanY, vanT | Glycopeptide | Antibiotic target alteration |
| MDC1 | vanY | Glycopeptide | Antibiotic target alteration |
|  | qacJ | Disinfecting agents and antiseptics | Antibiotic efflux |
| NCTC10389 | vanY, vanW | Glycopeptide | Antibiotic target alteration |
|  | qacJ | Disinfecting agents and antiseptics | Antibiotic efflux |
| NCTC8133 | vanY | Glycopeptide | Antibiotic target alteration |
|  | qacJ | Disinfecting agents and antiseptics | Antibiotic efflux |
| NCTC8140 | vanY | Glycopeptide | Antibiotic target alteration |
|  | qacJ | Disinfecting agents and antiseptics | Antibiotic efflux |
| S1 | vanY, vanT | Glycopeptide | Antibiotic target alteration |
| SheepZ001 | vanY | Glycopeptide | Antibiotic target alteration |
|  | qacJ | Disinfecting agents and antiseptics | Antibiotic efflux |
| *S*. *infantarius* |  |  |  |
| FDAARGOS_1019 | vanY, vanT | Glycopeptide | Antibiotic target alteration |
| *S*. *infantarius* subsp. *infantarius* | |  |  |
| CJ18 | vanY, vanT | Glycopeptide | Antibiotic target alteration |
| *S*. *lutetiensis* |  |  |  |
| FDAARGOS_1018 | vanY, vanT, vanW | Glycopeptide | Antibiotic target alteration |
| NCTC13774 | vanY, vanT, vanW | Glycopeptide | Antibiotic target alteration |
| FDAARGOS_1158 | vanY, vanT, vanW | Glycopeptide | Antibiotic target alteration |
| 033 | vanY, vanT, vanW | Glycopeptide | Antibiotic target alteration |
|  | tet(M) | Tetracycline | Antibiotic target protection |
|  | lnu(C) | Lincosamide | Antibiotic inactivation |
|  | erm(B) | Macrolide, lincosamide, streptogramin A, B | Antibiotic target alteration |
| NCTC11436 | tet(M) | Tetracycline | Antibiotic target protection |
|  | vanY, vanT, vanW | Glycopeptide | Antibiotic target alteration |
| NCTC8738 | vanY, vanT, vanW | Glycopeptide | Antibiotic target alteration |
| NCTC8796 | vanY, vanT, vanW | Glycopeptide | Antibiotic target alteration |
| *S*. *gallolyticus* |  |  |  |
| FDAARGOS_666 | ermT | Macrolide, lincosamide, streptogramin A, B | Antibiotic target alteration |
|  | tet(M), tet(45) | Tetracycline | Antibiotic target protection |
|  | vanY, vanT | Glycopeptide | Antibiotic target alteration |
| NCTC13773 | qacG | Disinfecting agents and antiseptics | Antibiotic efflux |
|  | vanY, vanT | Glycopeptide | Antibiotic target alteration |
| FDAARGOS_755 | qacG | Disinfecting agents and antiseptics | Antibiotic efflux |
|  | tet(M) | Tetracycline | Antibiotic target protection |
|  | vanY, vanT | Glycopeptide | Antibiotic target alteration |
| ICDDRB-NRC-S1 | qacJ | Disinfecting agents and antiseptics | Antibiotic efflux |
|  | vanY, vanT | Glycopeptide | Antibiotic target alteration |
| UCN34 | ermT | Macrolide, lincosamide, streptogramin A, B | Antibiotic target alteration |
|  | tet(45), tet(M) | Tetracycline | Antibiotic target protection |
|  | vanY, vanT | Glycopeptide | Antibiotic target alteration |
|  | qacG | Disinfecting agents and antiseptics | Antibiotic efflux |
| XH2168 | tet(45), tet(O/W/32/O) | Tetracycline | Antibiotic target protection |
|  | ANT(6)-Ia | Aminoglycoside | Antibiotic inactivation |
|  | ermB | Macrolide, lincosamide, streptogramin A, B | Antibiotic target alteration |
|  | lnuB | Lincosamide | Antibiotic inactivation |
|  | lsaE | Lincosamide, streptogramin, pleuromutilin | Antibiotic target protection |
|  | qacG | Disinfecting agents and antiseptics | Antibiotic efflux |
| *S*. *gallolyticus* subsp. *gallolyticus* | |  |  |
| ATCC43143 | vanY, vanT | Glycopeptide | Antibiotic target alteration |
|  | qacG | Disinfecting agents and antiseptics | Antibiotic efflux |
|  | tet(M) | Tetracycline | Antibiotic target protection |
| ATCCBAA-2069 | vanY, vanT | Glycopeptide | Antibiotic target alteration |
|  | tet(M) | Tetracycline | Antibiotic target protection |
| DSM16831 | vanY, vanT | Glycopeptide | Antibiotic target alteration |
|  | tet(M) | Tetracycline | Antibiotic target protection |
| TX20005 | vanY, vanT | Glycopeptide | Antibiotic target alteration |
|  | qacG | Disinfecting agents and antiseptics | Antibiotic efflux |
|  | tet(M) | Tetracycline | Antibiotic target protection |
| *S*. *macedonicus* |  |  |  |
| CIP105683 | vanY, vanT | Glycopeptide | Antibiotic target alteration |
|  | qacJ | Disinfecting agents and antiseptics | Antibiotic efflux |
| ACA-DC198 | vanY, vanT | Glycopeptide | Antibiotic target alteration |
|  | qacJ | Disinfecting agents and antiseptics | Antibiotic efflux |
| E37 | vanY, vanT | Glycopeptide | Antibiotic target alteration |
|  | qacJ | Disinfecting agents and antiseptics | Antibiotic efflux |
|  | tet(M), tet(S), tet(O) | Tetracycline | Antibiotic target protection |
|  | APH(3’)-IIIa, add(6) | Aminoglycoside | Antibiotic inactivation |
|  | ermB | Macrolide, lincosamide, streptogramin A, B | Antibiotic target alteration |
| *S*. *pasteurianus* |  |  |  |
| ATCC43144 | vanY, vanT | Glycopeptide | Antibiotic target alteration |
|  | tet(M), tet(45) | Tetracycline | Antibiotic target protection |
| NCTC13784 | vanY, vanT | Glycopeptide | Antibiotic target alteration |
|  | tet(M) | Tetracycline | Antibiotic target protection |
|  | APH(3’)-IIIa, add(6) | Aminoglycoside | Antibiotic inactivation |
|  | SAT-4 | Nucleoside antibiotic | Antibiotic inactivation |
|  | ermB | Macrolide, lincosamide, streptogramin A, B | Antibiotic target alteration |
| WUSP067 | AAC(6')-Ie-APH(2'')-Ia bifunctional protein | Aminoglycoside | Antibiotic inactivation |
|  | dfrF | Diaminopyrimidine | Antibiotic target replacement |
|  | lnuC | Lincosamide | Antibiotic inactivation |
|  | ermB | Macrolide, lincosamide, streptogramin A, B | Antibiotic target alteration |
|  | vanY, vanT | Glycopeptide | Antibiotic target alteration |
|  | tet(M), tet(45) | Tetracycline | Antibiotic target protection |
| WUSP070 | AAC(6')-Ie-APH(2'')-Ia bifunctional protein, ANT(6)-Ia, APH(3')-IIIa, add(6) | Aminoglycoside | Antibiotic inactivation |
|  | tet(O/W/32/O), tet(45) | Tetracycline | Antibiotic target protection |
|  | vanY, vanT, vanG | Glycopeptide | Antibiotic target alteration |
|  | lsaE | Lincosamide, streptogramin, pleuromutilin | Antibiotic target protection |
|  | lnuB | Lincosamide | Antibiotic inactivation |
|  | ermB | Macrolide, lincosamide, streptogramin A, B | Antibiotic target alteration |
|  | SAT-4 | Nucleoside antibiotic | Antibiotic inactivation |
| WUSP074 | APH(6)-Ia | Aminoglycoside | Antibiotic inactivation |
|  | vanY, vanT | Glycopeptide | Antibiotic target alteration |
|  | tet(O/W/32/O), tet(45), tet(O) | Tetracycline | Antibiotic target protection |
|  | ermB | Macrolide, lincosamide, streptogramin A, B | Antibiotic target alteration |
|  | lnuB | Lincosamide | Antibiotic inactivation |
|  | lsaE | Lincosamide, streptogramin, pleuromutilin | Antibiotic target protection |
| WUSP082 | APH(6)-Ia, APH(3’)=IIIa, add(6) | Aminoglycoside | Antibiotic inactivation |
|  | lsaE | Lincosamide, streptogramin, pleuromutilin | Antibiotic target protection |
|  | lnuG, lnuB | Lincosamide | Antibiotic inactivation |
|  | vanY, vanT | Glycopeptide | Antibiotic target alteration |
|  | tet(M), tet(O), tet(45) | Tetracycline | Antibiotic target protection |
|  | ermB | Macrolide, lincosamide, streptogramin A, B | Antibiotic target alteration |
|  | SAT-4 | Nucleoside antibiotic | Antibiotic inactivation |
| *S*. *alactolyticus* |  |  |  |
| LGM | APH(3')-IIIa, APH(6)-Ia, add(6) | Aminoglycoside | Antibiotic inactivation |
|  | lasE | Lincosamide, streptogramin, pleuromutilin | Antibiotic target protection |
|  | vanY, vanT | Glycopeptide | Antibiotic target alteration |
|  | lnuC, lnuB | Lincosamide | Antibiotic inactivation |
|  | SAT-4 | Nucleoside antibiotic | Antibiotic inactivation |
|  | qacG | Disinfecting agents and antiseptics | Antibiotic efflux |
| *S*. *ruminicola* |  |  |  |
| CNU_77-47 | vanY | Glycopeptide | Antibiotic target alteration |
| CNU_77-61 | vanY, vanT | Glycopeptide | Antibiotic target alteration |
|  | tet(M) | Tetracycline | Antibiotic target protection |
| CNU_G2 | Glycopeptide | Antibiotic target alteration |  |
|  | tet(M) | Tetracycline | Antibiotic target protection |
| CNU_G3 | Glycopeptide | Antibiotic target alteration |  |
|  | lnuC | Lincosamide | Antibiotic inactivation |

^*^The Resistance Gene Identifier (RGI, https://card.mcmaster.ca/analyze/rgi) web server was used to detect antimicrobial resistance genes in the complete genome sequence of representative SBSEC strains available in GenBank, focusing on perfect and strict hits.
